# A biosynthetic pathway for ornithine lipid formation dependent on a GH3 (Gretchen Hagen 3)-like enzyme in planctomycetes

**DOI:** 10.1016/j.jbc.2025.110634

**Published:** 2025-08-27

**Authors:** Lucero Yazmin Rivera-Najera, Enrique Manuel Cruz-Aguilar, Miguel Ángel Vences-Guzmán, Elena Rivas-Marin, Iván Ricardo Vega Valdez, Wendy Escobedo-Hinojosa, Ziqiang Guan, José Arcadio Farias-Rico, Damien P. Devos, Christian Sohlenkamp

**Affiliations:** 1 Centro de Ciencias Genómicas, Universidad Nacional Autónoma de México, Cuernavaca, Mexico; 2 Departamento de Genética, Facultad de Biología, Universidad de Sevilla, Seville, Spain; 3 Escuela Nacional de Ciencias Biológicas, Instituto Politécnico Nacional, Ciudad de México, Mexico; 4 Department of Biochemistry, Duke University School of Medicine, Durham, North Carolina, USA; 5 Institut Pasteur de Lille, Univ. Lille, CNRS, Inserm, CHU Lille, U1019 - UMR 9017 - CIIL - Center for Infection and Immunity of Lille, Lille, France

**Keywords:** ornithine lipids (OLs), amino lipids, Gretchen Hagen 3, *N*-acyl amino acids, membrane lipids

## Abstract

Amino lipids with acyloxyacyl structures, particularly ornithine lipids (OLs), are widespread in bacteria, but are absent from archaea and eukaryotes. In these lipids, an α-amino acid is *N*-acylated with a 3-hydroxy fatty acyl residue, and a secondary fatty acid is ester-bound to the hydroxyl group of the first fatty acid. Based on the presence of genes encoding the fatty acid transferases OlsBA or OlsF, involved in ornithine lipid (OL) synthesis, it has been estimated that approximately 50% of sequenced bacterial species can form OL. Here, based on genomic context, structure-based comparisons, and activity essays, we identified a novel pathway for OL synthesis in the planctomycete *Singulisphaera acidiphila*. This pathway initiates with OlsH, a GH3 (Gretchen Hagen 3)-like enzyme, that catalyzes the ATP-dependent formation of *N*-acyl ornithine. The α/β-hydrolase OlsI catalyzes the *O*-acyltransferase step. In the presence of OlsH and OlsI, we could reconstitute OL synthesis in a cell-free *Escherichia coli* protein extract. Structural modelling of OlsH revealed conserved GH3 nucleotide-binding motifs, and mutagenesis of serine residues validated their functional importance. Enzymatic assays demonstrated substrate specificity for ornithine and diverse fatty acids, and preference for Mg^2+^as cofactor. A phylogenomic analysis showed that bacterial GH3-homologues are widely distributed across diverse bacterial phyla. We conclude that bacterial GH3-like enzymes, such as OlsH described here, catalyze the initial step in OL synthesis in the planctomycete *S. acidiphila* and likely in other bacteria as well. These findings expand our understanding of bacterial lipid diversity and suggest evolutionary convergence in OL biosynthesis.

Bacterial membranes exhibit a wide variety of lipid structures beyond canonical glycerophospholipids such as phosphatidylethanolamine, phosphatidylglycerol, phosphatidylcholine, and cardiolipin (1). These alternative membrane lipids can be classified by their structure: Some, such as the sulfolipid sulfoquinovosyl diacylglycerol or the betaine lipid diacylglyceryl-*N*,*N*,*N*-trimethylhomoserine (DGTS), share a diacylglycerol (DAG) backbone (1, 2, 3), but others, such as hopanoids, ornithine lipids, or sphingolipids lack this DAG backbone (1, 2). While bacterial glycerophospholipid synthesis pathways are relatively well-explored, the biosynthesis, properties, and functions of these non-canonical membrane lipids frequently remain poorly understood.

Amino lipids containing an acyloxyacyl structure form a distinctive and important class of bacterial membrane lipids: α-amino acids are *N*-acylated with a 3-hydroxy fatty acyl residue, and a secondary fatty acid, also called “piggyback” fatty acid, is ester-bound to the hydroxyl group of the first fatty acid. Ornithine is the most common amino acid present in this type of lipid (1, 2, 4), but other headgroups that have been identified in these amino lipids are lysine, glycine, glutamine, serine-glycine, and taurine-ornithine (5). These lipids seem to be exclusive to bacteria and have not been found in archaea or eukaryotes. OLs are associated with environmental stress responses, including acid tolerance and temperature adaptation, and can modulate host immune responses (4, 6, 7, 8, 9, 10).

Although a diverse group of bacteria from different bacterial lineages has long been known to synthesize ornithine lipids (OLs), a pathway for OL formation was first discovered only approximately 2 decades ago by Geiger and co-workers using the nodule-forming α-proteobacterium *Sinorhizobium meliloti* as a model (11, 12). A decade later, the bifunctional enzyme OlsF was identified in *Serratia proteamaculans* (13). Based on the presence of genes encoding OlsB or OlsF homologs in bacterial genome sequences, we estimated that approximately 50% of sequenced bacterial species have the capacity to form OLs or other amino lipids. This number can be expected to increase further, as OL-forming bacteria lacking genes encoding OlsBA or OlsF homologs have been described (14, 15).

Previously, we identified and characterized the OL *N*-methyltransferase OlsG in the planctomycete *Singulisphaera acidiphila* (14). Interestingly, the *S. acidiphila* genome sequence lacks genes encoding identifiable OlsB or OlsF homologs, suggesting that a novel pathway might be operating. In the present study, we describe a third pathway for ornithine lipid synthesis. We combine biochemical assays, structural modeling, mutagenesis, and phylogenomic analysis to characterize this novel pathway. The acyl acid amido synthetase OlsH (Sinac_1599) catalyzes the formation of lyso-ornithine lipid (LOL) in an ATP-dependent reaction from ornithine and fatty acids, and the *O*-acyltransferase reaction required for OL formation from LOL is catalyzed by the protein OlsI (Sinac_1601) belonging to the α/β-hydrolase superfamily. Genes encoding bacterial GH3-like proteins, such as OlsH, are present in diverse bacterial phyla, where they may contribute to amino lipid synthesis. Our findings expand the known enzymatic diversity of bacterial membrane lipid biosynthesis and reveal a widespread yet underappreciated mechanism for membrane lipid synthesis.

## Results

### Discovery and functional validation of a third OL synthesis pathway in *S*. *acidiphila*

**Figure 1:**
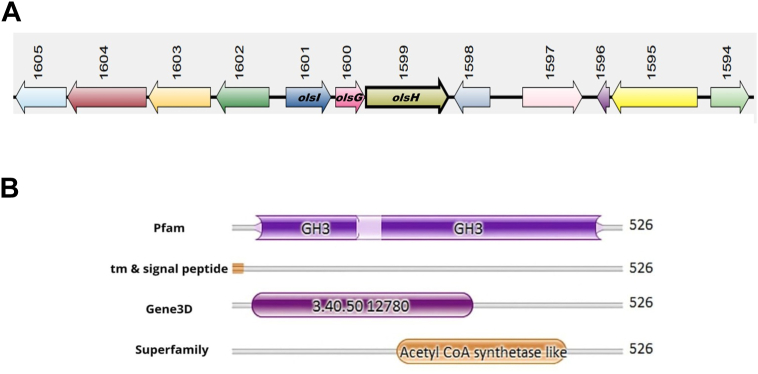
**Genomic context of the genes encoding enzymes involved in OL synthesis and modification, and analysis of the amino acid sequence of Sinac_1599.***A*, genomic context of the candidate genes Sinac_1599 and Sinac_1601.The gene that encodes Ornithine lipid *N*-methyltransferase OlsG (Sinac_1600) is flanked by the genes Sinac_1601 encoding OlsI and Sinac_1599 encoding OlsH. *B*, domain analysis of Sinac_1599.

OL can be synthesized *via* either the OlsBA pathway, first described in *S. meliloti*, or by the bifunctional enzyme OlsF, first described in *S*. *proteamaculans* (11, 12, 13). Earlier, we had described the *N*-methyltransferase OlsG, which catalyzes the *N*-methylation of the δ-nitrogen of ornithine in OL in *S*. *acidiphila* (14). An analysis of the *S. acidiphila* genome sequence revealed the absence of genes encoding OlsB or OlsF, suggesting the presence of a so-far unknown pathway. Interestingly, the gene encoding the OlsG *N*-methyltransferase (Sinac_1600) forms an operon with two additional genes, Sinac_1599 and Sinac_1601, in *S. acidiphila* (Fig. 1*A*). Sinac_1599 is annotated as an acyl-CoA synthetase of the GH3 (Gretchen Hagen 3) family (Fig. 1*B*), and Sinac_1601 as a protein of the α, β-hydrolase superfamily. Enzymes of the GH3 family are well-characterized in plants where they catalyze the aminoacylation of plant hormones such as indole acetic acid or jasmonic acid, forming jasmonic acid-isoleucine conjugates or indole acetic acid-aspartic acid conjugates (16, 17). They also catalyze a step in the isochorismate pathway for the biosynthesis of salicylic acid and are involved in the conjugation of auxinic herbicides (18). The bond formed between plant hormones and amino acids is an *N*-acyl bond, the same type of bond that is synthesized in the first step of OL synthesis. Sinac_1599, therefore, is a promising candidate gene to encode the missing enzyme responsible for the first step of OL synthesis in *S. acidiphila*. On the other hand, proteins with an α, β-hydrolase fold have been shown to have a diversity of functions (19), and a few α, β-hydrolase fold-containing enzymes have been characterized that can also work as acyltransferases (20, 21). We therefore considered that the gene Sinac_1601 might possibly encode the *O*-acyltransferase required for the second step of OL synthesis.

To test this hypothesis, the gene Sinac_1599 was PCR-amplified, cloned into an expression vector, and expressed in *Escherichia coli.* We studied the lipid profile of this *E. coli* strain under various growth conditions but were unable to identify *N*-acyl ornithine or a lyso-ornithine lipid (LOL). We then prepared a cell-free crude extract of the *E. coli* strain expressing Sinac_1599, added the putative substrates and [^14^C]-ornithine to the cell-free crude extract, and incubated it at 30 °C. The lipid fraction was then extracted and analyzed by thin-layer chromatography (TLC). A strong signal of a radioactive lipid migrating similar to purified LOL from *Agrobacterium tumefaciens* was observed (Fig. S1). The detected product is probably an *N*-acyl-ornithine formed with a non-hydroxylated fatty acid, and being less polar than a hydroxylated fatty acid, migrates slightly further away from the origin. No radioactive lipid was observed when the same assay was repeated with the cell-free protein extract of the empty vector control strain or with the extract from the *E. coli* strain expressing Sinac_1601 (Fig. S1). To confirm the results obtained by TLC, we set up several enzyme assays using the cell-free crude extract of the *E. coli* strain expressing Sinac_1599, this time adding cold ornithine. We then extracted and pooled the lipids and analyzed them by LC-MS. *N*-palmitoylated ornithine was detected in the *E. coli* strain expressing Sinac_1599 (Fig. S2).

**Figure 2:**
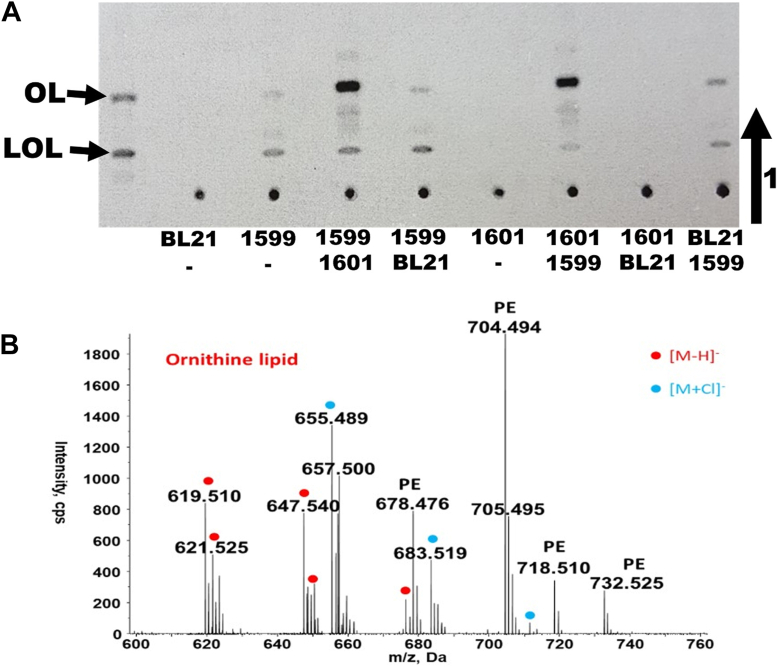
**Sinac_1599 and Sinac_1601 cause the formation of ornithine lipid *in vitro* in a cell-free protein extract of *E*. *coli*.***A*, thin-layer chromatography of lipids extracted from stepwise cell-free enzyme assays. Assays contained protein extract(s), [^14^C[-ornithine, 3-hydroxypalmitic acid, Mg^2+^, and ATP. The order of the extracts indicates the order in which they were added. 1: BL21(DE3).pLysS,pET17b, 2: BL21(DE3).pLysS,pET17b.1599, 3: BL21(DE3).pLysS,pET17b.1599 and BL21(DE3).pLysS,pET17b.1601, 4: BL21(DE3).pLysS,pET17b.1599 and BL21(DE3).pLysS,pET17b, 5: BL21(DE3).pLysS,pET17b.1601, 6: BL21(DE3).pLysS,pET17b.1601 and BL21(DE3).pLysS,pET17b.1599, 7: BL21(DE3).pLysS,pET17b.1601 and BL21(DE3).pLysS,pET17b, 8: BL21(DE3).pLysS,pET17b and BL21(DE3).pLysS,pET17b.1599. *B*, mass spectrometry analysis in negative ion mode of the lipids extracted from enzyme assays with Sinac_1599 and Sinac_1601, confirming the presence of ornithine lipids. Molecular ions corresponding to OLs, either as [M−H]^−^ or as [M+Cl]^−^ adducts, are labeled with dots.

**Figure 3:**
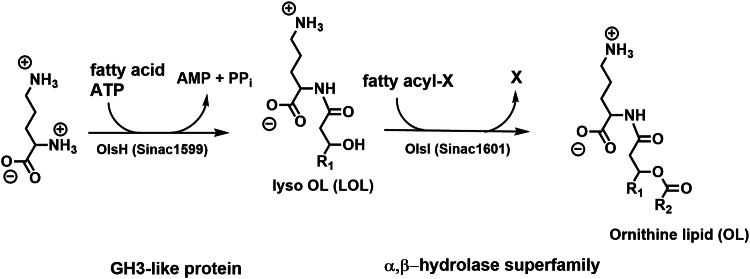
**Suggested pathway for OL synthesis in *S. acidiphila*.** X: unknown fatty acyl donor, for example, acyl-CoA or acyl-ACP.

Above, we had suggested that Sinac_1601 might encode the *O*-acyltransferase required for the second step of OL synthesis in *S. acidiphila*. To obtain evidence for this, a two-step assay was established. Protein-free crude extracts were prepared from an *E. coli* strain harboring the empty expression plasmid, from an *E. coli* strain expressing Sinac_1599, and from an *E. coli* strain expressing Sinac_1601. First, we set up an assay as described earlier for 30 min using one of the cell extracts (Fig. 2*A*, lanes 1, 2, 5), then a different cell-free protein extract was added for an additional 30 min (Fig. 2*A*, lanes 3, 4, 6, 7, 8). No labeled lipids were detected when Sinac_1601 and an empty plasmid control extract were present (Fig. 2*A*, lane 7). When Sinac_1599 and a control extract were present, the lipid pattern resembled that observed when only Sinac_1599 was present (lanes 4 and 8). Minor amounts of lipid migrating as purified OL were also observed in the same sample. This formation of small amounts of OL might be explained by an endogenous *O*-acyltransferase that can transfer fatty acids to LOL as an alternative substrate. When both Sinac_1599 and Sinac_1601 were present at the end of the assay (Fig. 2*A*, lanes 3 and 6), the most abundant radioactive compound was a lipid migrating as OL. To confirm the results obtained by TLC, we repeated enzyme assays containing Sinac_1599 and Sinac_1601 with cold ornithine, pooled several of the enzyme assays, extracted the lipids, and analyzed them by LC-MS. OLs were detected in the lipid extracts (Fig. 2*B*), confirming that Sinac_1601 encodes an *O*-acyltransferase responsible for the second step of OL synthesis. Therefore, we will refer for the rest of the present manuscript to Sinac_1599 as OlsH and Sinac_1601 as OlsI. These findings establish a third, biochemically distinct pathway for OL synthesis, composed of two non-homologous enzymes unrelated to OlsBA and OlsF. The proposed biosynthetic pathway for OL synthesis is shown in Figure 3.

### Deletion of OlsH in *S. acidiphila* abolishes trimethyl ornithine lipid formation

**Figure 4:**
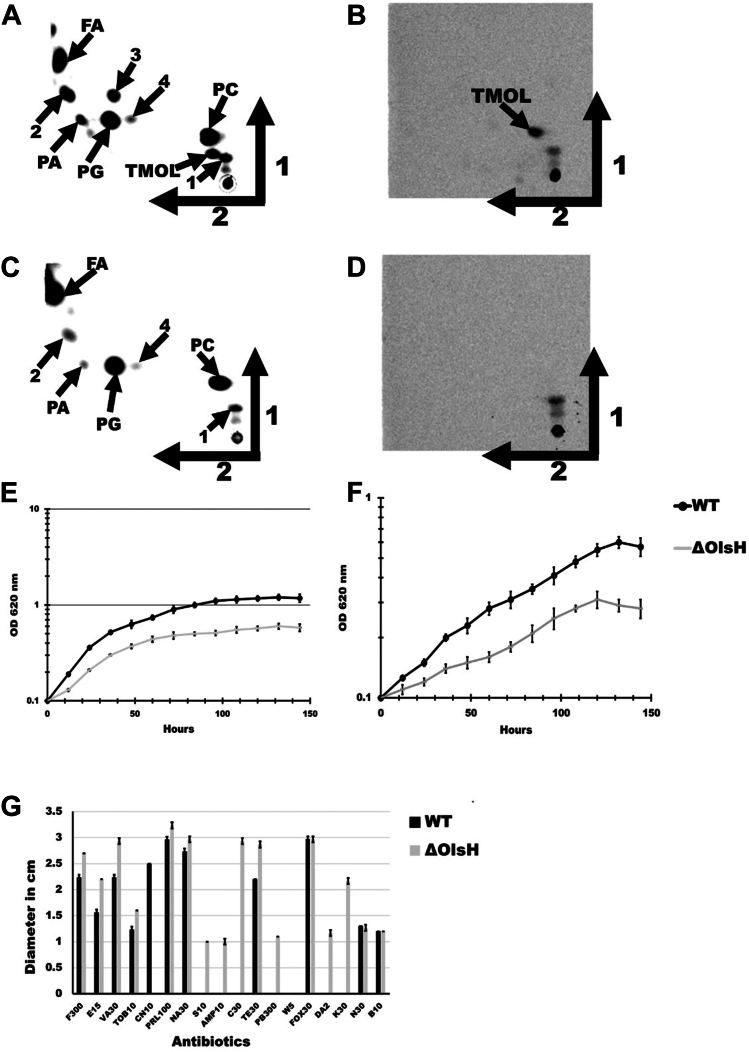
**A *S. acidiphila* mutant deficient in OlsH does not accumulate trimethylated OLs, grows more slowly in liquid media, and shows an increased susceptibility to several antibiotics.***S. acidiphila* wildtype (*A*, *B*) and OlsH-deficient mutant (*C*, *D*) were labeled for 24 h in the presence of [^14^C]-acetate (*A*, *C*) or [^14^C]-ornithine (*B*, *D*). Lipids were extracted and separated by two-dimensional thin-layer chromatography. Arrows indicate the first and second dimensions. PC: phosphatidylcholine, PG: phosphatidylglycerol; PA: phosphatidic acid, FA: fatty acids, TMOL: *N*-trimethyl ornithine lipid. The identities of lipid spots labeled with 1, 2, 3, and 4 are unknown. Growth of the *S. acidiphila* wildtype and the OlsH-deficient mutant was compared in liquid M31 medium (*E*) and liquid M31 medium without KH_2_PO_4_ supplementation (*F*). The experiment was repeated three times independently. Error bars indicate standard deviations. Bar graph showing the mean and standard deviation of the diameter of antibiotic-dependent sensitivity halos of the *S. acidiphila* wildtype and the OlsH-deficient mutant (*G*). F300-nitrofurantoin; E15-erythromycin; VA30-vancomycin; TOB10-tobramycin; CN10-gentamicin; PRL100-piperacillin; NA30-nalidixic acid; S10-streptomycin; AMP10-ampicillin; C30-chloramphenicol; TE30-tetracyclin; PB300-polymyxin B; W5-trimethoprim; FOX30-cefoxitin; DA2-clindamycin; K30-kanamycin; N30-neomycin; B10-bacitracin.

To confirm the role of OlsH in OL formation in *S. acidiphila* cells, we constructed an *S. acidiphila* mutant deficient in OlsH. If OlsH forms part of the OL synthesis pathway in *S. acidiphila*, the respective mutant should be unable to form LOL and therefore also no OLs, nor the *N*-methylated forms of OL. Moore *et al.* (15) had studied the membrane lipid composition of *S. acidiphila,* and they had shown that the major lipid classes were trimethyl OL (TMOL), phosphatidylcholine (PC), phosphatidylglycerol (PG), and phosphatidic acid (PA). Escobedo-Hinojosa had also detected the presence of free fatty acids and of a few additional lipids whose identity was unknown (14). We grew the *S. acidiphila* wildtype strain and the mutant deficient in OlsH in a modified M31 liquid medium (22) in the presence of either radiolabeled [^14^C]-acetate or [^14^C]-ornithine. In the acetate labeling of the wildtype strain, we detected the lipids TMOL, PC, PG, PA, and fatty acids, in addition to at least four lipids that could not be assigned (Fig. 4*A*). When labeling the same strain with [^14^C]-ornithine, in addition to the application spot, only one lipid was strongly labeled, while a second compound with less label was observed (Fig. 4*B*). According to the R_f_ value of the strongly labeled compound, we concluded that it is TMOL (14). When labeling with [^14^C]-acetate, fewer lipids were labeled in the OlsH mutant in comparison to the wildtype strain. The spot assigned as TMOL disappeared, along with another lipid spot whose identity we do not know (Fig. 4*C*), and which had not been observed in our earlier study (14). The absence of TMOL was also confirmed by labeling with [^14^C]-ornithine (Fig. 4*D*). We compared the growth of the wildtype and the OlsH-deficient mutant in liquid M31 medium (Fig. 4*E*) and in liquid M31 medium without KH_2_PO_4_ supplementation (Fig. 4*F*). This modified medium will still contain phosphate from the yeast extract, but it could be limiting for growth. The wildtype grows faster and reaches a higher final optical density in both media. We also compared the antibiotic resistance profile of both strains on M31 medium. The OlsH-deficient mutant was susceptible to six antibiotics to which the wildtype was resistant (streptomycin, ampicillin, chloramphenicol, polymyxin B, clindamycin, and kanamycin) and showed increased susceptibility to several other antibiotics. It was resistant to gentamicin, consistent with the use of a gentamicin cassette for mutant construction (Fig. 4*G*).

These results provide genetic evidence that OlsH catalyzes the initial step in OL synthesis in *S. acidiphila*, its absence blocking the formation of LOL, OL, and TMOL: We also establish *S. acidiphila* as a genetically tractable planctomycete, with our study presenting, to our knowledge, the first reported gene knockout in this genus.

### Structural modeling and comparative analysis reveal conserved GH3 features in OlsH

To further characterize OlsH (Sinac_1599), we analyzed its primary sequence and predicted its tertiary structure. The gene Sinac_1599, encoding OlsH, consists of 1581 bp and is annotated in NCBI as encoding an 'acyl-CoA synthetase (AMP-forming)/AMP-acid ligase II' of 526 amino acids. OlsH was predicted to be a soluble protein with a theoretical isoelectric point of 7.33 and a molecular weight of 58.75 kDa. Using the HMMER server and multiple domain prediction tools (23), we identified three conserved domains (Fig. 1*B*): a Gretchen Hagen 3 (GH3) domain (Pfam PF03321) formed by two overlapping regions, spanning residues 34 to 217 and 176 to 503; an N-terminal domain of the adenylating ANL superfamily (accession number 3.40.50.12780) in the Gene3D database, which spans residues 25 to 332; and an acetyl-CoA synthetase domain (Superfamily accession number 56801) spanning from residues 211 to 452 (Fig. 1*B*).

To evaluate the similarity with canonical GH3 enzymes, we decided to search first for OlsH homologues in the plant model *Arabidopsis thaliana*. One of the best hits with a confirmed function was the protein L-jasmonoyl amide synthetase (JAR1) (PBD 4EPL), also known as *At*GH3.11. Both proteins aligned over a stretch of 280 amino acids, with 21% identity and 36% similarity on an amino acid level. The protein JAR1 is responsible for conjugating the phytohormone jasmonic acid (JA) to the amino acid isoleucine (Ile) through an amide bond, a crucial step in activating the plant hormone (17). A BLASTp search with *At*GH3.11 revealed a total of 19 GH3-like proteins in *A. thaliana.* Even the most distant homologues could be aligned for a stretch of at least 400 amino acids, with 37% of identical amino acids and 55% of similar amino acids. For most of these 19 homologues, structures have been deposited in the PDB, and despite using different substrates, all of these present a strong structural conservation.

The OlsH sequence from *S. acidiphila* was aligned with sequences from selected GH3 proteins conjugating jasmonic acid from *A. thaliana*, *Oryza sativa*, *Solanum lycopersicum*, and *Vitis vinifera* (*At*GH3.11, *At*GH3.10, *Os*GH3.5, *Os*GH3.3, *Sl*JAR1, *Vv*GH3.7) (24, 25, 26, 27, 28) (Fig. S3). Despite low overall identity, a multiple sequence alignment using PROMALS3D (29) identified 83 highly conserved residues (conservation level 9), 22 probable α-helices, and 18 probable β-sheets. PSIPRED (30) predicted 23 possible α-helices and 16 possible β-sheets for the OlsH sequence. The characteristic nucleotide-binding motifs of GH3 proteins (S100SGTSQGRPK109, Y330GSSE334, and G397LYRYRLGD405, numbered according to the *At*GH3.11 protein) were identified in the alignment and, although divergent, could be recognized in OlsH. Residues from the OlsH amino acid sequence aligned with some residues involved in the jasmonate-binding site (F113, L117, L124, F125, G335, and W336), one residue implicated in isoleucine binding (S151), and with residues forming the hinge loop (L427, L429, S430, I431, I433, D434) (17, 18, 31) (Fig. S3).

**Figure 5:**
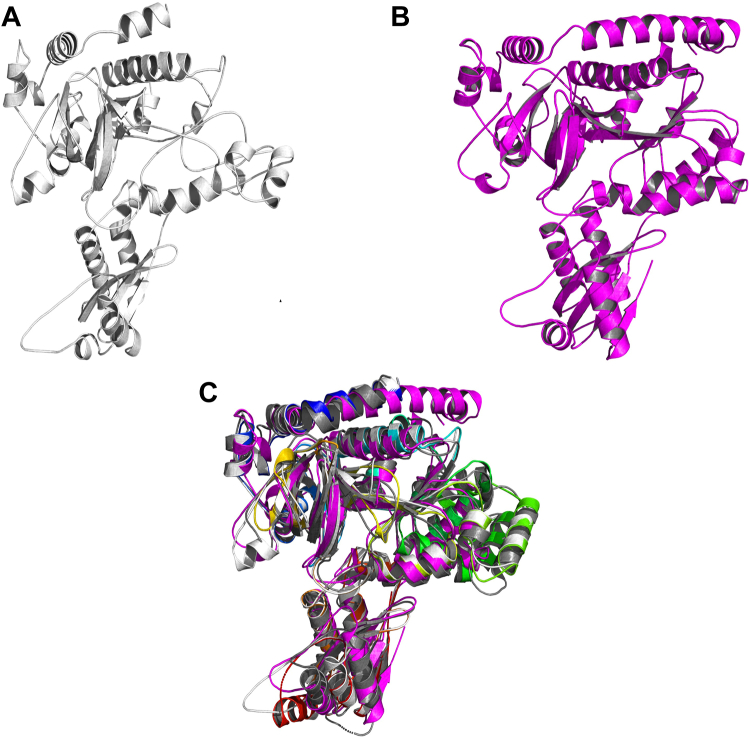
**Models of the three-dimensional structure of GH3.***A*, homology-based model of OlsH called 1599H, (*B*) OlsH model made with alpha-fold called 1599α, (*C*) Structural multiple alignment between the crystal structure of AtGH3.11 (4EPL, color scale from blue (N-terminal) to red (C-terminal)), the crystal structure of the AtGH3.12 protein (4EPM, *gray*), the 1599H model (*white*), and the 1599α model *(pink*).

To predict the three-dimensional structure of OlsH, we generated two models: The first by homology modeling using the Swiss-Model web server (template: *At*GH3.11, PDB 4EPL) (32), and another by deep learning *via* ColabFold (Fig. 5*B*) (AlphaFold2). The Swiss-Model (1599H) covered 492 out of the 526 amino acids (from residue 25–516) and featured 19 α-helices, 12 β-sheets, and 31 loops (Fig. 5*A*). Four regions of the protein, corresponding to approximately 5% of the entire chain, a total of 19 amino acids, could not be modeled: M1LEKVE6, G52NAT55, S367ETGEGE373, and S540SAGQF545. Additionally, 15% of the chain showed data with outlier values.

The AlphaFold model (1599α) covered the entire sequence and predicted a similar topology but with more compact loop regions (Fig. 5*B*) (Mirdita *et al.*, 2022). This model featured 21 α-helices, 17 β-sheets, and 38 loops. In both cases, the putative residues forming the nucleotide-binding motifs, the potential acyl group binding site residues, and the amino acid binding residues were located in the large N-terminal structural domain, except for the hinge loop residues, which were found in the small C-terminal structural domain. Structural validation using the SAVES v6.0 web server (33, 34, 35) and MolProbity (36) showed that model 1599α had superior geometric consistency (92.2% VERIFY3D pass rate) compared to model 1599H (73.3%).

**Figure 6:**
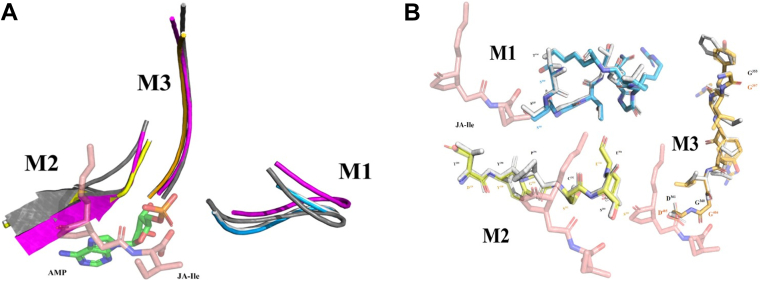
**Structural alignments of nucleotide-binding motifs.***A*, structural multiple alignment between the nucleotide-binding motifs of model 1599H (*white*), 1599α (*magenta*), and the nucleotide-binding motifs of crystal 4EPM (*gray*) and crystal 4EPL (*blue*: motif I, *yellow*: motif II, and *orange*: motif III). M1: nucleotide-binding motif I. M2: nucleotide-binding motif II. M3: nucleotide-binding motif III. (*B*) Structural alignment between the nucleotide-binding motifs of the 4EPL/AtGH3.11 crystal structure (*blue*, *yellow*, and *orange*) and the motifs of the 1599H model (*white*). JA-Ile: Jasmonoyl-Isoleucine. M1: nucleotide-binding motif one (*blue*). M2: nucleotide-binding motif two (*yellow*). M3: nucleotide-binding motif three (*orange*).

Structural superimposition of the OlsH models with the GH3 crystals (*At*GH3.11 and *At*GH3.12) (37, 38) using PDBeFold revealed a conserved global fold (Fig. 5*C*). When separately examining the structural alignment of the first, second, and third nucleotide-binding motifs of the OlsH models (T114SGTTQGATK123, T289YPCSE294, and G353MWSHLIGD361) with the corresponding nucleotide-binding motifs in the crystallized proteins 4EPL (S100SGTSQGRPK109, D329YGSSE334, and G397LYRYRLGD405) and 4EPM (S95SGTSGGANK104, T324YGSSE329, and G391LYLYRGD398), it was observed that the modeled motifs exhibited a three-dimensional structure and spatial arrangement similar to the motifs in the GH3 protein crystals (Fig. 6*A*). However, side-chain positioning in these motifs varied, especially in the AlphaFold model. Notably, model 1599H showed closer alignment of side-chain orientations with plant GH3 active sites, suggesting a better match for functional inference (Fig. 6*B*).

These results support the classification of OlsH as a functional GH3-like enzyme with structural conservation of key catalytic elements, despite its bacterial origin and low sequence identity with plant homologues.

### Substrate specificity and catalytic requirements of OlsH revealed by enzymatic assays and mutagenesis

**Figure 7:**
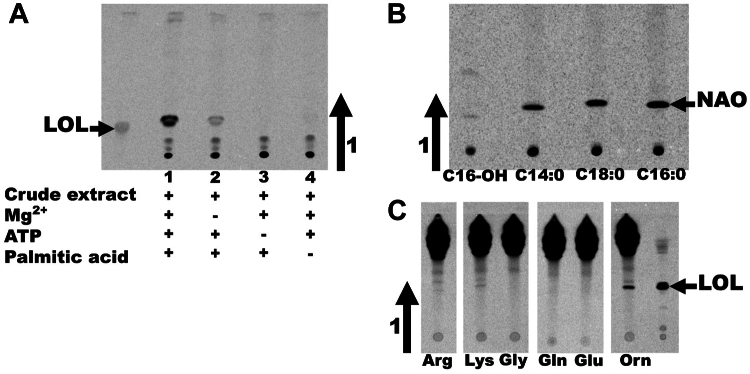
**Substrate specificity of OlsH.** Enzyme assays were set up under optimized conditions. *A*, LOL-purified lyso-ornithine lipid from *Agrobacterium tumefaciens*, 1- complete enzyme assay, 2- enzyme assay without Mg^2+^, 3- enzyme assay without ATP, 4- enzyme assay without palmitic acid. *B*, using radiolabeled [^14^C]-ornithine, the fatty acid substrate was varied: 1-hydroxypalmitic acid, 2-myristic acid, 3-stearic acid, 4-palmitic acid. *C*, using radiolabeled [^14^C]-palmitic acid, the amino acid substrate was varied. 1-arginine, 2-lysine, 3-glycine, 4-glutamine, 5-glutamic acid, 6-ornithine, 7-assay with ornithine using [^14^C]-ornithine instead of [^14^C]-acetate. LOL-lyso-ornithine lipid, NAO-*N*-acyl ornithine. The arrows indicate the directions of solvent migration during the TLC runs.

GH3 enzymes from plants use ATP to adenylate an acyl substrate in the first half-reaction. Nucleophilic attack by the α-amine of the amino acid leads to the release of AMP and the formation of an amino acid conjugate (39, 40). We considered that OlsH should function similarly and set up an assay using the cell-free crude extract containing OlsH, Mg^2+^, palmitic acid, and [^14^C]-ornithine. Adding all compounds resulted in a solid acyl-ornithine signal (Fig. 7*A*); however, omission of Mg^2+^ ions, ATP, or the fatty acid substrate strongly reduced or abolished enzymatic activity.

To assess substrate promiscuity, we tested different fatty acids: myristic acid, palmitic acid, stearic acid, or hydroxypalmitic acid. Strong acyl ornithine signals were detected with the three non-hydroxylated fatty acids, and only a very weak signal was detected when using hydroxypalmitate (Fig. 7*B*). Interestingly, when using hydroxypalmitate as substrate, in addition to LOL, a radioactive spot corresponding to OL was also detected, likely due to endogenous *E. coli* acyltransferase activity. Next, we wanted to vary the amino acid substrates, and as we had no access to the labeled amino acids, we tested amino acid specificity using [^14^C]-palmitate and the cold amino acids lysine, arginine, glycine, glutamic acid, or glutamine (Fig. 7*C*). OlsH showed a clear preference for ornithine, and a minor activity was observed with lysine and arginine and no acylation occurred with glutamic acid, glutamine, or glycine.

To probe the role of conserved residues of the identified nucleotide-binding motifs, two conserved serine residues, serine 115 and serine 293, from motif 1 (M1) and motif 2 (M2), respectively, that had been identified in the structural prediction studies, were selected, and single point variants of the nucleotide sequence were synthesized in which the codons encoding these serine residues were changed to codons encoding alanine residues. Enzymatic assays varying pH, ATP concentration, and divalent cations were performed with extracts containing the wild-type protein, the variants OlsH-S115A or OlsH-S293A, or an empty vector control extract.

**Figure 8:**
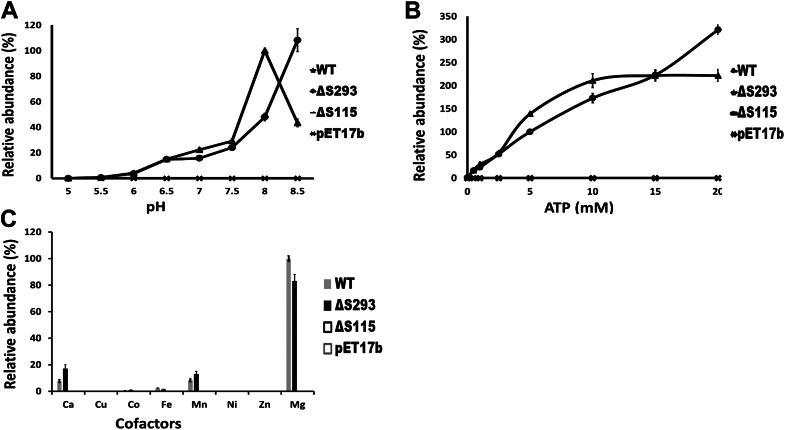
**Activity of OlsH and site-directed OlsH variants at different pH conditions, with different ATP concentrations, and with different cations.** 100% relative activity was defined as the activity of the standard assay containing 5 mM ATP, 10 mM Mg^2+^, [^14^C]-ornithine, and cell-free protein extract with OlsH. (*A*) pH dependence of OlsH activity, (*B*) ATP-dependence of OlsH activity, and (*C*) co-factor dependence of OlsH activity. No activity was detected in the empty vector control or the OlsH variant S115A-OlsH. Triangles represent the wildtype OlsH, and circles represent the variant S293A-OlsH. In *panel* C, *gray* is the wildtype OlsH, and *black* is the S293A-OlsH.

First, the pH of the assays was varied to evaluate at which pH the cell-free protein extract containing wildtype OlsH would show its highest acyl-ornithine synthase activity (Fig. 8*A*). Lipids were extracted from assay reactions, analyzed on TLC plates, and the acyl-ornithine lipid spots were quantified. With the wild-type enzyme, the highest activity was observed at pH 8. No activity was observed with the empty vector control and in the assays with variant OlsH-S115A. Variant OlsH-S293A showed a similar activity as the wildtype from pH 6 to pH 7.5, but an activity lower than the wild-type at pH 8 and an activity higher than the wild-type at pH 8.5 (Fig. 8*A*). Next, we varied the ATP concentrations of the assays to determine at which concentration OlsH would exhibit the highest activity. We used concentrations from 0 mM to 20 mM ATP in the assays. With the wildtype OlsH, we observed an increase in activity up to 10 mM ATP, and OlsH activity did not increase further at higher ATP concentrations. Again, no activity was observed in the empty vector control and with variant OlsH-S115A. In contrast, variant OlsH-S293A showed an increase throughout the tested concentration range, with the highest activity observed at 20 mM ATP (Fig. 8*B*). Finally, the divalent cation present in the assay was varied to determine whether other cations could be utilized in the reaction catalyzed by OlsH. Eight different cations were used at a final concentration of 10 mM each (CaCl_2_, CoCl_2_, CuSO_4_, FeSO_4_, MnCl_2_, NiCl_2_, ZnSO_4_, and MgCl_2_) (Fig. 8*C*). OlsH activity was observed in the presence of calcium (Ca^2+^), cobalt (Co^2+^), iron (Fe^2+^), manganese (Mn^2+^), and magnesium (Mg^2+^) (Fig. 8*C*). The highest activity was observed in the presence of magnesium. Enzyme activities in the presence of calcium and manganese were clearly lower, but in a similar range. In the presence of cobalt and iron, only very minor OlsH activities were observed. Again, no enzyme activities were observed with mutant OlsH-S115A and with empty vector control. Mutant OlsH-S293A showed a lower activity than the wildtype with magnesium, but a slightly increased activity with calcium and manganese compared to the wildtype enzyme.

Our results demonstrate that OlsH prefers ornithine and non-hydroxylated fatty acids as substrates, requires ATP and Mg^2+^, and is catalytically dependent on residue S115. Residue S293 contributes to activity modulation but is not essential for catalysis. This characterization reinforces the functional similarity between OlsH and canonical GH3 enzymes.

### Widespread distribution and phylogenetic divergence of GH3-like enzymes in bacteria

After identifying that OlsH and OlsI form a new pathway for OL synthesis in bacteria, we sought to learn about the phylogenetic distribution of genes encoding bacterial OlsH homologues. A BLASTp search using the OlsH amino acid sequence as query was made against the database of bacterial genomes. This search yielded more than 7500 hits, almost 90% of which were in the phylum Bacteroidota, and the rest mainly in the phyla Pseudomonadota and Planctomicetota, and a few others, indicating a broad phylogenetic distribution. Within the Bacteroidota, most hits were found in the classes Bacteroidia, Flavobacteriia, Cytophagia, and Sphingobacteriia. Within the Pseudomonadota, hits were mainly located in the α-proteobacteria and in other classes. Interestingly, some genomes encode multiple GH3-like proteins, suggesting a potential functional diversification within a single organism.

To explore relationships between GH3 proteins, we constructed a phylogenetic tree including selected sequences from plant GH3 proteins and bacterial GH3 homologs.

**Figure 9:**
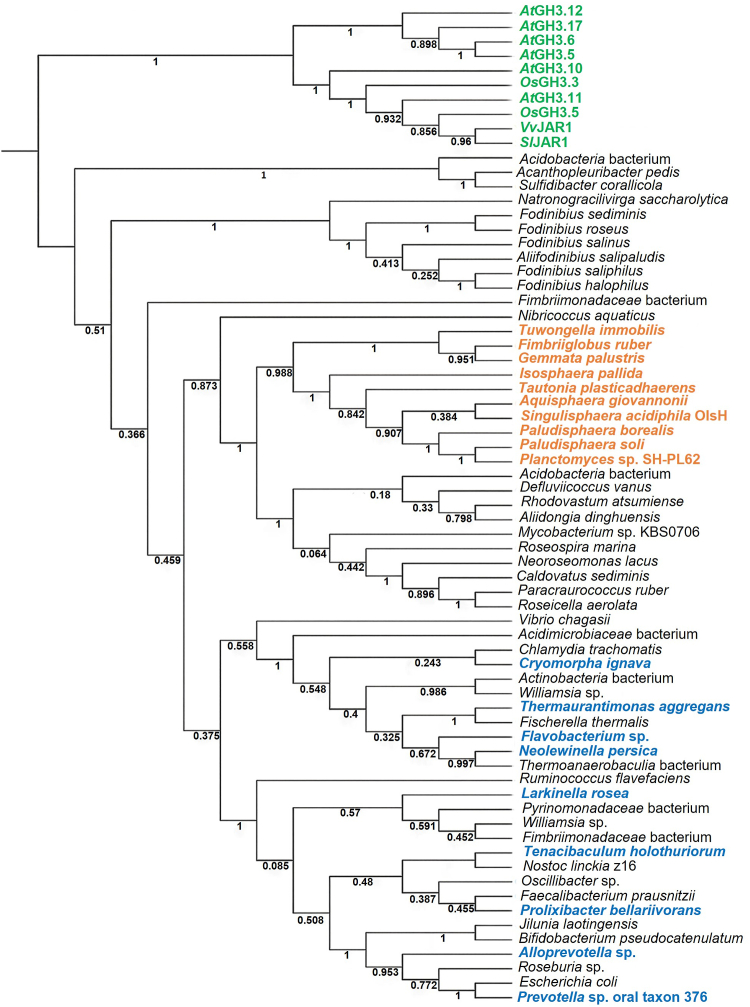
**Phylogenetic tree to study the possible distribution of OlsH homologs.** Cladogram of plant GH3 protein family sequences and bacterial amino acid sequences homologous to OlsH (Sinac_1599), generated using the MEGA 11 software (64). The full names and accession numbers for each sequence are listed in the Methods section. The values on the internal branches of the phylogenetic tree represent bootstrap values. Plant sequences are labeled in *green*, planctomycete sequences are labeled in *orange*, and sequences from the CFB group are labeled in *blue*.

Plant sequences formed a distinct outgroup (Fig. 9, green), further divided into two major clades corresponding to their substrates. The bacterial sequences formed different clades, which in some cases were exclusive or at least enriched for a specific taxonomic group, for example, in the cases of Acidobacteria, Balneolaeota, planctomycetes, or proteobacteria. Notably, in the lower part of the tree, the clades are not particularly enriched in specific taxonomic groups. Homologues from planctomycetes clustered closely with *S. acidiphila* OlsH, and in all cases, OlsH and OlsI form part of a single operon, supporting the hypothesis that they likely share substrate specificity for ornithine (Fig. 9, orange). OlsI homologues can also be detected in the genomes of the CFB group containing OlsH homologues (Fig. 9, blue), but both genes are not in a single operon. OlsH homologues outside the planctomycetes may exhibit specificity for different amino acids and catalyze the formation of other *N*-acyl amino acids.

Our findings suggest that GH3-like enzymes are widespread across bacteria and may have diversified to catalyze the synthesis of a broad range of amino lipid types. The functional implications of this diversity remain to be elucidated, but the evolutionary conservation of core structural features supports a common catalytic mechanism.

## Discussion

Amino lipids containing an acyloxyacyl structure are common in bacteria and represent a unique class of membrane components absent from archaea and eukaryotes. The amino acid headgroups described are glycine, glycine-serine, ornithine, ornithine-taurine, lysine, and glutamine. The most common amino acid in this type of lipid is ornithine; in this case, the complete membrane lipid is called ornithine lipid (OL). The presence of OLs contributes to stress resistance in bacteria. They have been shown to confer improved growth at increased temperatures and low pH conditions in *Rhizobium tropici* (10). Formation of OLs in *E. coli* (which normally does not form OLs) *via* heterologous expression improved pH resistance and enhanced biomass production under phosphate-limiting conditions (41). Recently, it has been demonstrated that OLs act as partial TLR4 agonists and NLRP3 activators (7).

Based on genome analysis, it has been estimated that approximately 50% of sequenced bacterial species can form OL. The first pathway for OL synthesis was described in *S. meliloti,* where formation of OLs is catalyzed by two enzymes, the *N*-acyltransferase OlsB and the *O*-acyltransferase OlsA (4, 9, 11, 12). Later, the bifunctional acyltransferase OlsF with two acyltransferase domains was described, each responsible for one step in OL synthesis (1, 13). The OlsBA pathway is present in several α- and β-proteobacteria, as well as in a few γ-proteobacteria, and some actinobacteria. OlsF exhibits a different distribution, being present in a few γ-proteobacteria, δ-proteobacteria, ε-proteobacteria, and bacteria of the CFB (Cytophaga-Flavobacterium-Bacteroidetes) group. These unmodified OLs can be functionalized after synthesis by hydroxylation, *N*-methylation, and taurine transfer, allowing adaptation of membrane properties in the absence of new synthesis (6, 10, 14, 42, 43, 44).

Here, we identify a third pathway for OL synthesis in the plantomycete *S. acidiphila*, which is formed by the GH3-like enzyme OlsH and the *O*-acyltransferase OlsI. The amino acid sequence of OlsH is unrelated to the sequences of OlsB or the *N*-acyltransferase domain of OlsF, and the amino acid sequence of OlsI is unrelated to OlsA or the *O*-acyltransferase domain of OlsF. The fact that bacteria have evolved three different ways to synthesize OLs suggests that OLs confer a selective advantage to bacteria under various conditions, particularly during adaptation to oligotrophic environments.

We have frequently heterologously expressed genes encoding enzymes responsible for membrane lipid synthesis using *E. coli* as host cells, resulting in most cases in the successful detection of the activity of the respective enzyme by observing the end products. Here, to our surprise, OlsH was not causing the synthesis of *N*-acyl-ornithine inside living *E. coli* cells. However, we could detect activity in cell-free crude extracts when adding the enzyme’s substrates, ornithine, free fatty acids, ATP, and Mg^2+^. We believe that, upon expression in *E. coli*, at least one of the substrates is not accessible to OlsH inside the cells. A lack of ornithine should not be the problem, because we have successfully shown that expression of the OL synthase OlsF causes OL formation in *E. coli* cells (13, 14, 41). ATP and magnesium ions should also be available, but a possible explanation is that little free fatty acid is found in *E. coli,* as fatty acid synthesis in this bacterium is tightly coupled to phospholipid synthesis (45, 46, 47, 48). On the other hand, in our earlier study describing the OL *N*-methyltransferase OlsG, we noted several lipids from *S. acidiphila* migrating in our TLC system as free fatty acids (14), which suggests that larger amounts of free fatty acids are present in *S. acidiphila* than in *E. coli*.

We observed that in the OlsH (Sinac_1599) enzyme assays, small amounts of a lipid migrating as OL were sometimes detected, even in the absence of OlsI (Sinac_1601). Probably, an endogenous *E. coli* protein can catalyze the *O*-acylation reaction required for OL formation. We would expect a lysophosphatidic acid acyltransferase or an sn-glycerol 3-phosphate acyltransferase to catalyze this reaction. A certain promiscuity of lyso-phosphatidic acid acyltransferases has been observed in some bacteria (49, 50).

To confirm that OlsH forms part of the OL synthesis pathway in *S. acidiphila*, we constructed a deletion mutant deficient in the *olsH* gene and compared the wildtype lipid composition to the lipid composition of the OlsH mutant. The mutant construction in itself was a major achievement, as a few mutants have been constructed in other genera belonging to the planctomycetes, and to our knowledge, this is the first mutant reported in the genus *Singulisphaera*. In the wildtype, the only OL detected was TMOL, and this lipid was absent in the mutant. We also observed that another unknown lipid, which was present in the wildtype, disappeared in the OlsH mutant. This lipid was not labeled with ornithine, so its absence should not be a direct effect of the absence of OlsH, but rather an indirect effect related to the absence of TMOL. We also observed radiolabel in a compound migrating close to the origin in our thin-layer chromatography experiments, both in the mutant and in the wildtype. As the compound is labeled, the molecule must be derived from ornithine and retain its carboxyl group. We speculate that it may be citrulline, which can be formed from ornithine by the enzyme ornithine transcarbamylase. The absence of OLs in the OlsH-deficient mutant strongly affected growth in M31 medium and in M31 medium without phosphate supplementation, and the changes in the membrane composition also affected the antibiotic resistance profile of the mutant strain.

A total of 19 GH3-like proteins has been identified in *A. thaliana*. Between them, even the most distant homologues can be aligned for a stretch of at least 400 amino acids, with 37% of identical amino acids and 55% of similar amino acids. Structures of many of these homologues have been deposited in the PDB, and all of these present a strong structural conservation. GH3 proteins share a common tertiary fold defined by a large N-terminal domain and a smaller C-terminal domain typical of adenylating enzymes. Staswick *et al.* identified structural similarity between the *A. thaliana* GH3 and the firefly luciferase-like superfamily of proteins (51, 52). The active site is located at the interface of the two domains (18). OlsH is annotated as a GH3-like protein, but the sequence conservation on an amino acid level with the well-characterized plant enzymes is relatively low. Still, its sequence can be modelled onto the structures published for plant GH3 proteins, and also AlphaFold models a similar structure. Consistent with this, OlsH, as the plant GH3 proteins, catalyzes the formation of an amide bond, and here we show that OlsH is responsible for the synthesis of *N*-acyl ornithine.

Despite the relatively low sequence conservation between plant GH3 proteins and OlsH, the three nucleotide-binding motifs characteristic of GH3 proteins can be identified. We selected two conserved serine residues and synthesized two mutated versions of OlsH, each encoding a serine-to-alanine change. One of these mutant proteins had lost its activity under all conditions tested, indicating the importance of this specific serine residue. The second mutant protein exhibited activity similar to the wildtype protein under most conditions, although some minor differences were observed between the two versions of the protein, indicating that it likely plays a role in OlsH activity but that it is not essential for its function.

All adenylate-forming enzymes are reported to be Mg^2+^-dependent; however, in at least one case, Mn^2+^ has been shown to substitute for Mg^2+^ (53). The requirement for magnesium or manganese has been described for the GH3 protein OsGH3-8 from *O. sativa* (39). OlsH activity was highest in the presence of magnesium ions, and much lower activity was also observed in the presence of calcium ions and bivalent manganese ions.

OlsH does not require acyl-carrier protein (ACP) nor CoA as an acyl donor, which could facilitate its use in technological applications. Interestingly, several GH3 proteins from *A. thaliana* have been crystallized, and although they use a wide range of acyl and amino acyl substrates, they present a very similar structure (18). *N*-acyl amino acids could have application as biosurfactants, and the structural similarity between proteins with different substrate preferences could allow for the design of alternative enzymes using new substrate combinations, similar to what was suggested for bacterial CfaL enzymes, which were modified and designed to synthesize a diverse array of amides (54).

We have shown here that OlsH catalyzes the first step in OL synthesis in *S. acidiphila*, a bacterium of the planctomycetes. Genes encoding bacterial GH3-like proteins are widespread in certain bacterial groups. They are most abundant in the CFB group, and interestingly, in this group, it has been shown that OlsB and OlsF homologues are responsible for OL formation, making it unlikely that the GH3 homologues are responsible for *N*-acyl ornithine formation. Similar to OlsBA and OlsF homologs that catalyze the synthesis of other amino lipids, we think that several of the homologues will also use other amino acids as substrates. *N*-acyl amino acids have been described in a wide range of Bacteroidetes and in gut-inhabiting members of the Clostridia (55, 56). For example, bacteria of the genus *Olivibacter*, belonging to the Sphingobacteria, form *N*-acyl tyrosine, and their genomes encode bacterial GH3 homologues. Further studies are needed to understand the potentially diverse functions of bacterial GH3-like proteins.

In conclusion, this study describes a third, structurally and mechanistically distinct pathway for OL synthesis. The identification of OlsH and OlsI offers opportunities to explore the ecological role and possible biotechnological applications of amino lipids.

## Experimental procedures

### Strains and plasmids used in this study

*S. acidiphila* (DSM 18658T) was purchased from the Deutsche Sammlung von Mikroorganismen und Zellkulturen GmbH (DSMZ). It was grown at 28 °C in a modified M31 liquid medium (22) containing per liter of distilled water 0.1 g of KH_2_PO_4_, 20 ml of Hutner's basal salt solution (57), 1 g of *N*-acetylglucosamine, 0.1 g of peptone, 0.1 g of yeast extract, adjusted to pH 5.6. We replaced ammonium molybdate with equimolar amounts of sodium molybdate in Hutner's basal salts solution. In some growth experiments comparing the wildtype and the OlsH-deficient mutant, KH_2_PO_4_ was omitted from the medium. Antibiotic resistance profiles were determined as described earlier (58). *E*. *coli* strain DH5α was used for cloning, whereas *E. coli* strain BL21(DE3).pLysS was used for the overexpression of Sinac_1599 (OlsH) and Sinac_1601 (OlsI). *E. coli* strains were grown at 30 °C in LB medium (59). When required, antibiotics were added to *E. coli* cultures in the following final concentrations: 100 mg/liter carbenicillin and 10 mg/liter chloramphenicol.

### DNA manipulations, cloning of *S. acidiphila* candidate genes, and *S. acidiphila* mutant construction

Recombinant DNA techniques were performed according to standard protocols (59). Oligonucleotide primer sequences are listed in Table S1.

Genomic DNA from *S. acidiphila* was isolated from bacterial 1.5-ml cultures using a DNA isolation kit for cells and tissues (Roche). ORFs of Sinac_1599 (OlsH) and Sinac_1601 (OlsI) were amplified from genomic DNA by PCR using Phusion DNA polymerase (Thermo). Amplified PCR products and the expression vector pET17b (60) were digested with the respective restriction enzymes, and the ORFs were ligated into pET17b. Nucleotide sequences of the constructs were confirmed by Sanger sequencing. The resulting plasmids (Table S2) were transformed into *E. coli* BL21(DE3).pLysS. DNA fragments encoding the mutated versions of OlsH-S115A and OlsH-S293A were synthesized by the company T4OLIGO (Irapuato). The ORFs were flanked by NdeI-BamHI sites to facilitate the cloning into the expression plasmid. In case of OlsH-S115A, the triplet 343AGC345 was changed to GCC. In case of OlsH-S293A, the triplet 877TCC879 was changed to GCC.

To construct the plasmid (Table S2) to delete the *S. acidiphila olsH* gene (sinac_1599), 899 and 900 bp upstream and downstream fragments of the target gene, respectively, were amplified by PCR from genomic DNA using the primer pairs listed in Table S1. The upstream and downstream fragments were digested with EcoRI/BamHI and BamHI/PstI, respectively, and cloned into pEX18Tc by three-way ligation (61). Finally, the gentamicin resistance gene amplified from the pBBRMCS5 plasmid was subsequently cloned as a BamHI fragment between the two flanking regions (62). Nucleotide sequences of the constructs were confirmed by Sanger sequencing. The resulting plasmid (pDV199) was transformed into *S. acidiphila* by electroporation. Fresh electrocompetent cells were prepared from 400 ml of an *S. acidiphila* culture at an OD600 of 0.4 in M31 liquid medium. The cells were washed twice with 100 and 50 ml of ice-cold double-distilled sterile water, and once with 2 ml of ice-cold 10% glycerol. Then, the pellet was resuspended in 400 μl of ice-cold 10% glycerol, and aliquots of 100 μl were made. An aliquot of competent cells was dispensed into 0.1-mm gapped electroporation cuvettes along with 0.5-1 μg of purified plasmid and electroporated with EC3 pulse (3.0 kV). Electroporation was performed with a BioRad Micropulser. Electroporated cells were immediately recovered in 1 ml of cold M31 liquid medium and incubated at 28 °C for 2 h with shaking. The cells were then plated onto M31 medium plates supplemented with 50 μg/ml cycloheximide and 20 μg/ml gentamicin, and incubated at 28 °C until colony formation. Colonies were segregated onto fresh selection plates and genotyped by PCR using the primers Out sinac_1599 fwd and Out sinac_1599 rv.

### *In vivo* labeling of *S. acidiphila*

The lipid compositions of bacterial strains were determined following labelling with [1-^14^C]-acetate (Amersham Biosciences), or [^14^C]-ornithine (PerkinElmer) was added. Cultures (2 ml) of wild-type and mutant strains were inoculated from pre-cultures grown in the same medium. After the addition of 1 μCi of [^14^C]-acetate (60 mCi mmol^−1^) or 1 μCi of [^14^C]-ornithine (56 mCi mmol^−1^) to each culture, the cultures were incubated for 16 h. The cells were harvested by centrifugation, washed with 500 μl of water, and resuspended in 100 μl of water. Lipid extracts were obtained according to Bligh and Dyer (63). Aliquots of the lipid extracts were spotted on high-performance TLC silica gel 60 (Merck, Poole, UK) plates and were separated into two dimensions using chloroform/methanol/water (140:60:10, v/v) as a mobile phase for the first dimension and chloroform/methanol/glacial acetic acid (130:50:20, v/v) for the second dimension. To visualize the membrane lipids, two-dimensional TLC plates were exposed to a PhosphorImager screen (Amersham Biosciences). The individual lipids were quantified using ImageQuant software (Amersham Biosciences).

### Enzyme assays for OlsH (Sinac_1599)

To determine the activity of the Sinac_1599-encoded protein, *in vitro* assays were performed at 30 °C using a protein extract that had been filtered three times with a 0.2 μm pore Amicon filter. For the reaction, micelles of the fatty acid with Triton X-100 were prepared by mixing 100 μl of 0.1% Triton X-100 with 10 μl of 200 mM fatty acid and drying the mixture under vacuum. Then, 10 μl of 1 M Tris buffer pH 7.9 was added, mixed thoroughly, and sonicated for 20 min. To this micelle mixture, [^14^C]-ornithine (56 mCi/mmol, 0.1 mCi/ml) at 35 μM was added, along with 4 μl of 200 mM ATP, 4 μl of 100 mM MgCl_2_, and 100 μl of protein extract (protein concentration 1.5 mg/ml). The final reaction volume was 200 μl; the final concentrations of Tris and MgCl_2_ were 50 mM and 2 mM, respectively. After 30 min of incubation, the reaction was stopped by adding 500 μl of methanol and 250 μl of chloroform. The lipid fraction was extracted as previously described (63). The lipid extract was analyzed by TLC. Lipid spots were quantified using a Storm 820 PhosphorImager and ImageQuant software (Amersham Biosciences).

### Acylation assay in the presence of different amino acids

Micelles were prepared with [^14^C]-palmitic acid (60 mCi/mmol, Amersham). ATP, protein extract, and one of the various tested amino acids were added to the micelle mixture, in a final volume of 25 μl. After 20 min of incubation, the reaction was stopped by adding methanol:chloroform (2:1). The lipid fraction was then extracted and analyzed by TLC. Lipid spots were quantified using a Storm 820 PhosphorImager and ImageQuant software (Amersham Biosciences).

### Lyso-ornithine lipid acylation assay, dependent on the Sinac_1601-encoded protein

*In vitro* assays to determine OlsI (Sinac_1601) activity were performed at 30 °C. Micelles were prepared as previously described, using 3-hydroxypalmitic acid. 4 mM ATP, 35 μM [^14^C]-ornithine, and protein extract containing the OlsH, and/or OlsI were added to the micelle mixture in a final volume of 200 μl. After 1 h of incubation, the reaction was stopped by adding 500 μl methanol and 250 μl chloroform. The lipid fraction was extracted as previously described (63), and the lipid extract was analyzed by TLC. Lipid spots were quantified using a Storm 820 PhosphorImager and ImageQuant software (Amersham Biosciences).

### Liquid chromatography/tandem mass spectrometry analysis of lipid samples

Various enzyme assays were pooled, and the lipids extracted according to Bligh and Dyer (63). Normal phase LC-ESI MS of the lipid extracts was performed using an Agilent 1200 Quaternary LC system coupled to a high-resolution TripleTOF5600 mass spectrometer (Sciex). Chromatographic separation was performed on an Ascentis Silica HPLC column, 5 μm, 25 cm × 2.1 mm (Sigma-Aldrich). Elution was achieved with mobile phase A, consisting of chloroform/methanol/aqueous ammonium hydroxide (800:195:5, v/v/v), mobile phase B, consisting of chloroform/methanol/water/aqueous ammonium hydroxide (600:340:50:5, v/v/v/v) and mobile phase C, consisting of chloroform/methanol/water/aqueous ammonium hydroxide (450:450:95:5, v/v/v/v), over a 40 min-long run, performed as follows: 100% mobile phase A was held isocratically for 2 min and then linearly increased to 100% mobile phase B over 14 min and held at 100% B for 11 min. The mobile phase composition was then changed to 100% mobile phase C over 3 min and held at 100% C for 3 min, and finally returned to 100% A over 0.5 min and held at 100% A for 5 min. The LC eluent (with a total flow rate of 300 μl/min) was introduced into the ESI source of the high resolution TF5600 mass spectrometer. MS and MS/MS were performed in negative ion mode, with the full-scan spectra being collected in the *m/z* 200 to 2000 range. The MS settings are as follows: Ion spray voltage (IS) = −4500 V (negative ion mode), Curtain gas (CUR) = 20 psi, Ion source gas 1 (GS1) = 20 psi, De-clustering potential (DP) = −55 V, and Focusing Potential (FP) = −150 V. Nitrogen was used as the collision gas for tandem mass spectrometry (MS/MS) experiments. Data analysis was performed using Analyst TF1.5 software (Sciex).

### Protein alignments and modeling of protein structures

The *At*GH3.11 protein was one of the first hits when searching with the Sinac_1599 sequence (OlsH) for proteins with a confirmed function. A multiple sequence alignment (MSA) was performed between the amino acid sequence of OlsH and the proteins *At*GH3.11 (OAP07631.1) and *At*GH3.10 (OAO98077.1) from *A. thaliana*, *Os*GH3.5 (XP_025881172.1) and *Os*GH3.3 (XP_015622064.1) from *O. sativa* L. ssp. *japonica, Sl*JAR1 (XP_010327002.1) from *S. lycopersicum*, and *Vv*JAR1/*Vv*GH3.7/JAR1 from *V. vinifera* (XP_010661892.1) using the PROMALS3D web server to determine whether the OlsH sequence might be related to GH3 proteins involved in jasmonoyl-isoleucine formation. PROMALS3D not only aligns the input sequences but also searches for homologous sequences to create a more robust alignment (29). The PROMALS3D server first performs an initial alignment using a weighted sum-of-pairs scoring function with BLOSUM62 scores to generate a series of pre-aligned groups that are relatively distant from each other. Subsequently, it generates a second alignment by selecting a representative sequence from each group to conduct a PSI-BLAST search, retrieving homologous sequences (using the UNIREF90 database), and predicting secondary structures with PSIPRED. Additionally, a pairwise hidden Markov model (HMM) of profile-profile alignments with predicted secondary structures and alignments with homologs possessing three-dimensional structures is generated to obtain a probabilistic consistency scoring function. This function is constrained by the probabilities obtained for each residue from the HMM and the constraints derived from alignments with homologs that have three-dimensional structures. Finally, the representative sequences are progressively aligned based on the consistency scoring function, and the pre-aligned groups are combined with the alignment of the representative sequences to form the final multiple sequence alignment (29). PROMALS3D includes a conservation scale ranging from Level 6 to Level 9, where six indicates a conserved residue and nine denotes a highly conserved residue. It provides two consensus sequences: The first is a consensus sequence based on residues conserved throughout the multiple sequence alignment (MSA), while the second consensus sequence is a prediction of the probable secondary structures that the sequences may exhibit (29). The recommended parameters by the authors were used: Identity threshold above which fast alignment is applied, 0.6; Weight for constraints derived from sequences, 1; Weight for constraints derived from homologs with structures, 1.5; Weight for constraints derived from input structures, 1.5; Weight for user-defined constraints, 1.5.

### Generation of three-dimensional models of the Sinac_1599 (OlsH) amino acid sequence

The first model was created using the homology method, based on the crystal structure *At*GH3.11 (PDB ID: 4EPL) retrieved from the Protein Data Bank (PDB, https://www.rcsb.org/), *via* the Swiss-Model web server (https://swissmodel.expasy.org/), and was designated as 1599H. The 4EPL crystal was used because it had a resolution of 2.01 Å, presented a good validation report by PDB (Rfree: 0.212; Clashscore = 4; Ramachandra outliers = 0 and Sidechain outliers = 0.2%) and 85% of the peptide chain was determined. The second three-dimensional model was produced using machine learning methods on GitHub's ColabFold v1.5.2: AlphaFold2 using MMseqs2 and was designated as 1599α. In both cases, the amino acid sequence of OlsH (Sinac_1599) was used, and the processes were executed with default parameters. Three prediction cycles were performed, and five models were generated. From these, the model with the best overall score in MolProbity was selected, considering quality parameters such as the clashscore, residues in favored regions of the Ramachandran plot (>98%) and the absence of stereochemical outliers. Subsequently, the three-dimensional models of the OlsH sequence were evaluated using the MolProbity server and SAVES v6.0 (Overall Quality Factor > 70%).

### Multiple structural alignment between the three-dimensional model of the Sinac_1599 (OlsH) and plant GH3 family proteins

A multiple structural alignment was performed using the crystal structures of the AtGH3.11 protein (4EPL) and the GH3.12 protein from *A*. *thaliana* (4EPM) obtained from the Protein Data Bank (PDB), the three-dimensional model 1599H, and the three-dimensional model 1599α using the PDBeFold protein structure comparison service from the European Bioinformatics Institute. This analysis aimed to determine whether the generated models have a conformation similar to the crystal structures and to assess whether the potential binding motifs present in the models align with the nucleotide-binding motifs of the crystal structures. The program PyMOL was used to visualize the structures. Prior to visualization, the rotation-translation matrix (coordinates to align each crystal and model to the same point) generated by PDBeFold was added to each ∗.pdb file.

### Construction of a phylogenetic tree to visualize the distribution and diversity of bacterial GH3-like proteins

The cladogram was constructed using the amino acid sequence of Sinac_1599 from *S. acidiphila* (OlsH); sequences of selected GH3 proteins from plants (10): *At*GH3.5, *At*GH3.6, *At*GH3.10, *At*GH3.11, *At*GH3.12, *At*GH3.17, *Os*GH3.5, *Os*GH3.3, *Sl*JAR1, and *Vv*JAR1; and selected amino acid sequences homologous to the OlsH sequence from different bacterial groups: Acidobacteria (6): *Acidobacteria* bacterium (MCL6490827.1), *Acidobacteria* bacterium (MCB1049466.1), *Acanthopleuribacter pedis* (WP_207862478.1), *Pyrinomonadaceae* bacterium (MBC7916061.1), *Sulfidibacter corallicola* (WP_237381738.1), and *Thermoanaerobaculia* bacterium (MCK6693358.1); Actinobacteria (6): *Acidimicrobiaceae* bacterium (MBT69625.1), *Actinobacteria* bacterium (MSZ02679.1), *Bifidobacterium pseudocatenulatum* (MZM33252.1), *Mycobacterium* sp. KBS0706 (TSD83934.1), *Williamsia* sp. (MBE7171429.1), and *Williamsia* sp. (MBE7172738.1); Armatimonadetes (2): *Fimbriimonadaceae* bacterium (MBC8046834.1) and *Fimbriimonadaceae* bacterium (MBC8047200.1); Balneolaeota (7): *Aliifodinibius salipaludis* (WP_095604961.1), *Fodinibius halophilus* (WP_165264994.1), *Fodinibius roseus* (WP_073059004.1), *Fodinibius salinus* (WP_170245548.1), *Fodinibius saliphilus* (WP_138429308.1), *Fodinibius sediminis* (WP_142715891.1), and *Natronogracilivirga saccharolytica* (WP_210511454.1); CFB (10): *Alloprevotella* sp. (MBF0945495.1), *Cryomorpha ignava* (WP_163285928.1), *Flavobacterium* sp. (MBC7744751.1), *Larkinella rosea* (WP_124878921.1), *Neolewinella persica* (WP_020567887.1), *Prevotella* sp. oral taxon 376 (WP_107620398.1), *Prolixibacter bellariivorans* (WP_025865098.1), *Tenacibaculum holothuriorum* (WP_086031110.1), and *Thermaurantimonas aggregans* (WP_124397661.1); Chlamydia (1): *Chlamydia trachomatis* (CRH27110.1); Cyanobacteria (2): *Fischerella thermalis* CCMEE 5319 (PMB17261.1) and *Nostoc linckia* z16 (PHK35909.1); Firmicutes (5): *Faecalibacterium prausnitzii* (VDR34392.1), *Jilunia laotingensis* (WP_262433767.1), *Oscillibacter* sp. (MCM1030387.1), *Roseburia* sp. (MCM1312461.1), and *Ruminococcus flavefaciens* (MCM1530980.1); Planctomycetes (10): *S. acidiphila* Sinac_1599 (OlsH) (AGA25978.1), *Aquisphaera giovannonii* (WP_148592567.1), *Gemmata palustris* (WP_210663547.1), *Isosphaera pallida* (WP_013565005.1), *Fimbriiglobus ruber* (WP_088260918.1), *Paludisphaera borealis* (WP_076344918.1), *Paludisphaera soli* (WP_165245946.1), *Planctomyces* sp. SH-PL62 (WP_068418529.1), *Tautonia plasticadhaerens* (QDV33767.1), and *Tuwongella immobilis* (VTR97332.1); Proteobacteria (10): *Aliidongia dinghuensis* (WP_189051126.1), *Caldovatus sediminis* (WP_188898990.1), *Defluviicoccus vanus* (WP_190261508.1), *E. coli* (MBL1009069.1), *Neoroseomonas lacus* (WP_229681057.1), *Paracraurococcus ruber* (WP_200305704.1), *Rhodovastum atsumiense* (WP_150038394.1), *Roseicella aerolata* (WP_226611664.1), *Roseospira marina* (WP_150061320.1), and *Vibrio chagasii* (WP_137406803.1); Verrucomicrobia (1): *Nibricoccus aquaticus* (ATC64126.1).

A total of 69 amino acid sequences were used to construct the cladogram, with plant GH3 protein family sequences selected as the out-group. The phylogenetic tree was built using the Maximum Likelihood method, employing the Le-Gascuel (LG) evolutionary model with a Gamma distribution and invariant sites (G + I), comprising two categories (+G, parameter = 2.0520; [+I], 1.86% sites). The analysis was replicated 1000 times using the Bootstrap method. Plant GH3 sequences are shown in green, plantomycete GH3-homologues are shown in orange, and CFB group GH3-homologs are shown in blue.

Sequences were retrieved from the SyntTax database and the NCBI protein database *via* BLASTp. The selection of the evolutionary model, as well as the construction and editing of both cladograms, were performed using the MEGA 11 software.

## Data availability

All data used in the article are available from the corresponding author upon reasonable request.

## Supporting information

This article contains supporting information.

## Conflict of interest

The authors declare that they have no conflicts of interest with the contents of this article.
