# Supplementary material for: A biosynthetic pathway for ornithine lipid formation dependent on a GH3 (Gretchen Hagen 3)-like enzyme in planctomycetes
Source: J Biol Chem. 2025 Aug 27;301(10):110634. doi: 10.1016/j.jbc.2025.110634 (PMC12493201; doi:10.1016/j.jbc.2025.110634)
Supplement: Supporting Figures and Tables [file mmc1.docx]

**Supplementary information for the article:**

**A biosynthetic pathway for ornithine lipid formation dependent on a GH3 (Gretchen Hagen 3)-like enzyme in planctomycetes**

Lucero Yazmin Rivera-Najera^1,7^, Enrique Manuel Cruz-Aguilar^1^, Miguel Ángel Vences-Guzmán^1^, Elena Rivas-Marin^2,3^, Iván Ricardo Vega-Valdez^4^, Wendy Escobedo-Hinojosa^1,8^, Ziqiang Guan^5^, José Arcadio Farias-Rico^1^, Damien P. Devos^6^, Christian Sohlenkamp^1*^

^1^Centro de Ciencias Genómicas, Universidad Nacional Autónoma de México, Cuernavaca, Mexico.

^2^Departamento de Genética, Facultad de Biología, Universidad de Sevilla, Seville, Spain

^3^Consejo Superior de Investigaciones Científicas (CSIC), Spain.

^4^Escuela Nacional de Ciencias Biológicas, Instituto Politécnico Nacional, Ciudad de México, Mexico

^5^Department of Biochemistry, Duke University School of Medicine, Durham, North Carolina, USA.

^6^Institut Pasteur de Lille, Univ. Lille, CNRS, Inserm, CHU Lille, U1019 - UMR 9017 - CIIL - Center for Infection and Immunity of Lille, F-59000 Lille, France

^7^Current Address: Instituto de Biotecnología, Universidad Nacional Autónoma de México, Cuernavaca, Mexico

^8^Current Address: Unidad de Química en Sisal, Yucatán. Facultad de Química, Universidad Nacional Autónoma de México, Mexico

Keywords: Ornithine lipids (OLs), amino lipids, Gretchen Hagen 3, *N*-acyl amino acids

**^*^**Corresponding author: Christian Sohlenkamp, Tel. 52-777-3131697; Fax. 52-777-3175581; E-mail: [chsohlen@ccg.unam.mx](mailto:chsohlen@ccg.unam.mx).

**Supplementary Figure S1.**

**
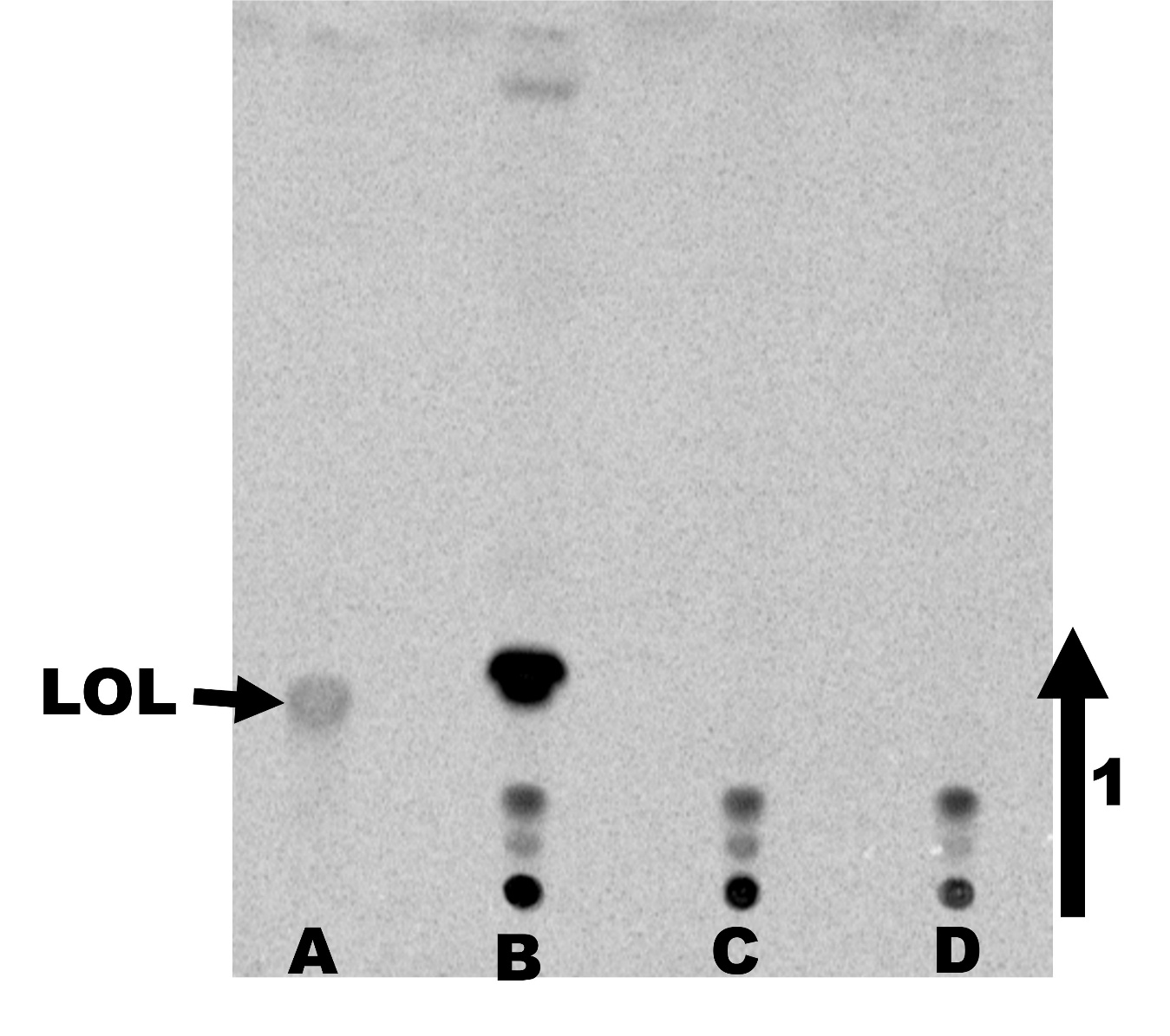
**

**Supplementary Figure S1. In the presence of Sinac_1599, *N*-acyl ornithine formation can be detected.** (A) Purified lyso-ornitine lipid (LOL), (B) enzyme assay using a protein extract containing Sinac_1599, (C) enzyme assay using a protein extract from an *E. coli* vector control strain, (D) enzyme assay using a protein extract containing Sinac_1601. The arrow indicates the direction of solvent migration.

**Supplementary Figure S2.**

**
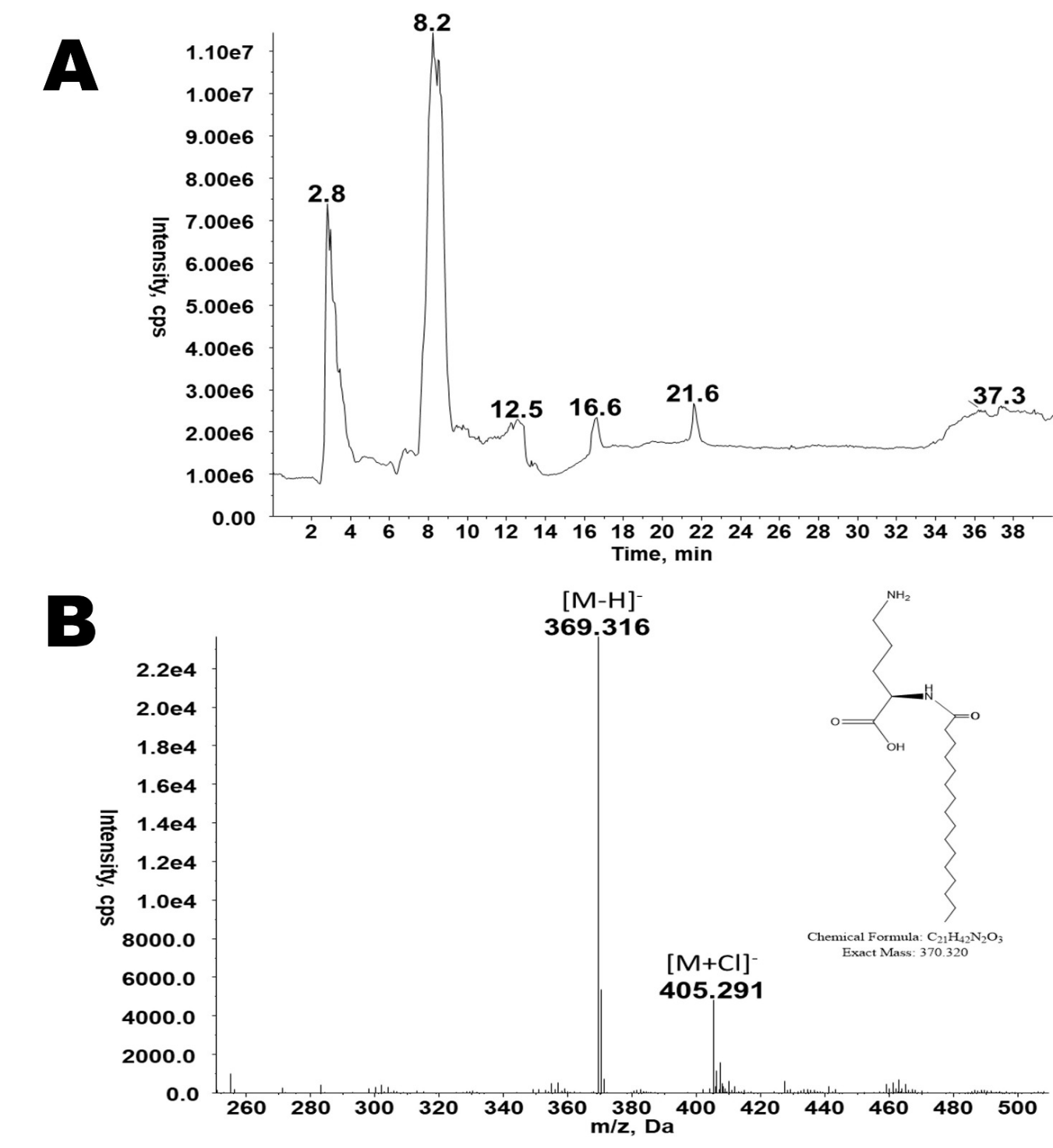
**

**Supplementary Figure S2. Detection of the reaction product *N*-palmitoyl ornithine by LC-MS. (A)** Total ion chromatogram of the LC-MS analysis in the negative ion mode of the lipid extract from the pooled enzyme assay using a cell-free protein extract from *E. coli* BL21(DE3).pLysS.pET17b.1599. (B) Negative ion mass spectrum (averaged from the spectra acquired between 21.4 min and 21.8 min) showing the [M-H]^-^ ion at *m/z* 369.316 for *N*-palmitoyl ornithine whose chemical structure is shown as an inset in panel B.

**Supplementary Figure S3.**

**
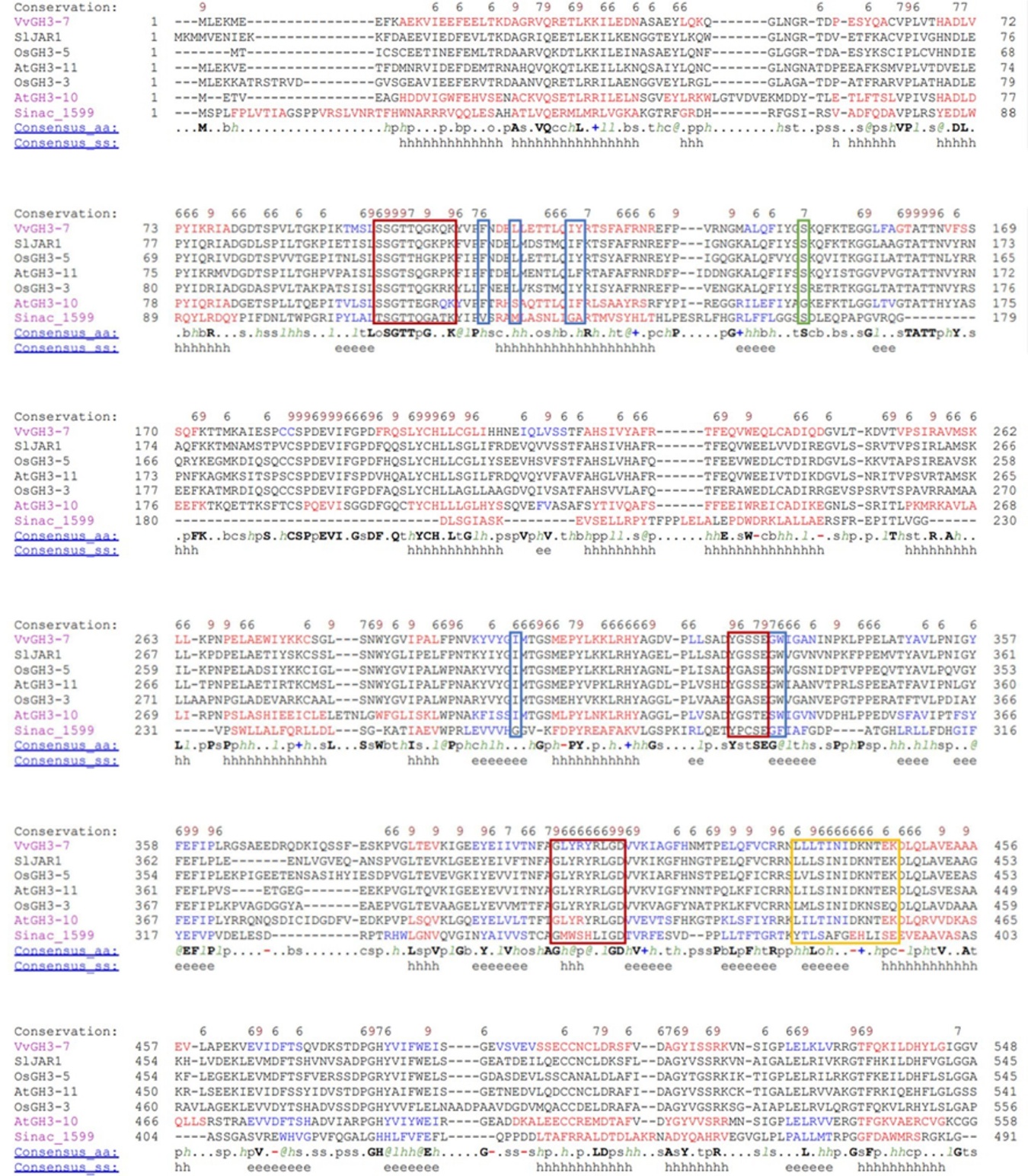
**

**Supplementary Figure S3: Multiple sequence alignment between Sinac_1599 and plant GH3 proteins**

Multiple sequence alignment between the amino acid sequence of OlsH (Sinac_1599) and the sequences of GH3 proteins involved in JA-Ile formation, performed using the PROMALS3D web server (Pei et al., 2008). Red boxes enclose the nucleotide-binding motifs. Blue boxes enclose the conserved residues involved in acyl group binding. The green box encloses the conserved residues involved in amino acid binding. The yellow box encloses the conserved residues that form the hinge loop. The line labeled 'Consensus_aa:' refers to the consensus amino acid sequence generated by the server, and 'Consensus_ss:' refers to the predicted secondary structures (Consensus_ss:). Uppercase bold amino acids indicate conserved residues. e: β-sheet (blue letters); h: α-helix (red letters); @: aromatic residue (Y, H, W, F); h: hydrophobic residue (W, F, Y, M, L, I, V, A, C, T, H); l: aliphatic residue (I, V, L); b: bulky residue (E, F, I, K, L, M, Q, R, W, Y); c: charged residue (D, E, K, R, H); o: alcohol residue (S, T); p: polar residue (D, E, H, K, N, Q, R, S, T); t: tiny residue (A, G, C, S); s: small residue (A, G, C, S, V, N, D, T, P); +: positively charged residue (K, R, H); -: negatively charged residue (D, E); 5: minimum conservation; 9: maximum conservation.

**Supplementary Table 1. Oligonucleotide primers used in this study.** All sequences are written in 5´to 3´direction

| **Name** | **Sequence (5’ – 3’)** | **Purpose** |
| --- | --- | --- |
| **Sinac_1599F** | ACTCGC**CATATG** AGC CCGTTATTTCCGCTGGTCACGATCGCC | Forward primer for the amplification of the *sinac_1599* gene and subsequent cloning into pET17b (NdeI site in bold) |
| **Sinac_1599R** | ACTCGC**GGATCC**CTATCCCGGTGGCAACTC CCGCTCGATCCA | Reverse primer for the amplification of the *sinac_1599* gene and subsequent cloning into pET17b (BamHI site in bold) |
| **Sinac_1601F** | ACTCGC**CATATG**AATCCAACCGTGCGGAAGTTCTTCGACCTC | Forward primer for the amplification of the *sinac_1601* gene and subsequent cloning into pET17b (NdeI site in bold) |
| **Sinac_1601R** | ACTCGC**GGATCC**TCATCTGTTCTGATGTGAGATGGTCGACGT | Forward primer for the amplification of the *sinac_1601* gene and subsequent cloning into pET17b (BamHI site in bold) |
| **LFR_Sinac1599 fwd** | ATTA**GAATTC**GAGAACGATCAGGTCATCTC | Forward primer for the amplification of the left flanking region of the *sinac_1599* gene and subsequent cloning into pEX18Tc (EcoRI site in bold) |
| **LFR_Sinac1599 rv** | TTA**GGATCC**CTCAGAAACTTTCCTTCACG | Reverse primer for the amplification of the left flanking region of the *sinac_1599* gene and subsequent cloning into pEX18Tc (BamHI site in bold) |
| **RFR_Sinac1599 fwd** | TTA**GGATCC**CCGGGATAGGCCTGGGTGC | Forward primer for the amplification of the right flanking region of the *sinac_1599* gene and subsequent cloning into pEX18Tc (BamHI site in bold) |
| **RFR_Sinac1599 rv** | AT**CTGCAG**GTCTGCGGTGACGAGTGTG | Reverse primer for the amplification of the right flanking region of the *sinac_1599* gene and subsequent cloning into pEX18Tc (PstI site in bold) |
| **Gm pBBRMCS5 fwd** | TCA**GGATCC**GTTGACATAAGCCTGTTCGG | Forward primer for the amplification of the gentamicin resistance marker from pBBRMCS5 and subsequent cloning into pEX18Tc containing the left and right *sinac_1599* flanking regions (BamHI site in bold) |
| **Gm pBBRMCS5 rv** | CAT**GGATCC**TTAGGTGGCGGTACTTGGG | Reverse primer for the amplification of the gentamicin resistance marker from pBBRMCS5 and subsequent cloning into pEX18Tc containing the left and right *sinac_1599* flanking regions (BamHI site in bold) |
| **Out_Sinac1599 fwd** | AACCGGCCCTCTTGATCTGG | Forward primer for the verification of the mutant strain *S. acidiphila* Δ *sinac_1599* |
| **Out_Sinac1599 rv** | TGTGGCGGATTCGAGTCTGG | Reverse primer for the verification of the mutant strain *S. acidiphila* Δ *sinac_1599* |

**Supplementary Table 2. Plasmids used in this study**

| Name | Purpose | Reference |
| --- | --- | --- |
| pET17b | Expression plasmid for use in *E. coli* BL21(DE3).pLysS. | (Studier,1991) |
| pET17b.1599 | pET17b-with gene *sinac_1599* cloned as NdeI/BamHI-fragment. | This study |
| pET17b.1601 | pET17b-with gene *sinac_1601* cloned as NdeI/BamHI-fragment. | This study |
| pET17b. OlsH-S115A | pET17b-with gene *olsH-S115A* cloned as NdeI/BamHI-fragment. | This study |
| pET17b. OlsH-S293A | pET17b-with gene *olsH-S293A* cloned as NdeI/BamHI-fragment. | This study |
| pEX18Tc | Tc^R^; *oriT*^+^ *sacB*^+^, gene replacement vector with MCS from pUC18 | (Hoang et al., 1998) |
| pBBRMCS5 | Mobilisable shuttle and expression vector, pBBR1MCS series, used to amplify gentamicin resistance marker. Gm^R^. | (Kovach et al., 1995) |
| pDV199 | 899 bp upstream (EcoRI/BamHI) and 900 bp downstream (BamHI/PstI) of *sinac_1599* gene from *S. acidiphila* flanking a gentamicin resistance gene cloned into pEX18Tc EcoRI/PstI. Gm^R^, Tc^R^ | This study |

References for Supplementary Information:

Hoang, T. T., Karkhoff-Schweizer, R. R., Kutchma, A. J., and Schweizer, H. P. (1998) A broad-host-range Flp-FRT recombination system for site-specific excision of chromosomally-located DNA sequences: application for isolation of unmarked *Pseudomonas aeruginosa* mutants Gene 212, 77-86 10.1016/s0378-1119(98)00130-9

Kovach, M. E., Elzer, P. H., Hill, D. S., Robertson, G. T., Farris, M. A., Roop, R. M., 2nd et al. (1995) Four new derivatives of the broad-host-range cloning vector pBBR1MCS, carrying different antibiotic-resistance cassettes Gene 166, 175-176 10.1016/0378-1119(95)00584-1

Pei, J., Kim, B. H., and Grishin, N. V. (2008) PROMALS3D: a tool for multiple protein sequence and structure alignments Nucleic Acids Res **36**, 2295-2300 10.1093/nar/gkn072

Studier, F. W. (1991) Use of bacteriophage T7 lysozyme to improve an inducible T7 expression system J Mol Biol 219, 37-44 10.1016/0022-2836(91)90855-z
